# Neural network based integration of assays to assess pathogenic potential

**DOI:** 10.1038/s41598-023-32950-5

**Published:** 2023-04-13

**Authors:** Mohammed Eslami, Yi-Pei Chen, Ainsley C. Nicholson, Mark Weston, Melissa Bell, John R. McQuiston, James Samuel, Erin J. van Schaik, Paul de Figueiredo

**Affiliations:** 1 Netrias, LLC, 1162 Gateway Drive, Annapolis, MD 21409 USA; 2 Special Bacteriology Reference Laboratory, Bacterial Special Pathogens Branch, Division of High-Consequence Pathogens and Pathology, Centers for Disease Control and Prevention, Atlanta, GA 30333 USA; 3 Department of Microbial Pathogenesis and Immunology, Texas A&M Health Science Center, Bryan, TX 77807 USA; 4 Department of Veterinary Pathobiology, Texas A&M University, College Station, TX 77843 USA

**Keywords:** Computational biology and bioinformatics, Microbiology, Pathogenesis, Mathematics and computing

## Abstract

Limited data significantly hinders our capability of biothreat assessment of novel bacterial strains. Integration of data from additional sources that can provide context about the strain can address this challenge. Datasets from different sources, however, are generated with a specific objective and which makes integration challenging. Here, we developed a deep learning-based approach called the neural network embedding model (NNEM) that integrates data from conventional assays designed to classify species with new assays that interrogate hallmarks of pathogenicity for biothreat assessment. We used a dataset of metabolic characteristics from a de-identified set of known bacterial strains that the Special Bacteriology Reference Laboratory (SBRL) of the Centers for Disease Control and Prevention (CDC) has curated for use in species identification. The NNEM transformed results from SBRL assays into vectors to supplement unrelated pathogenicity assays from de-identified microbes. The enrichment resulted in a significant improvement in accuracy of 9% for biothreat. Importantly, the dataset used in our analysis is large, but noisy. Therefore, the performance of our system is expected to improve as additional types of pathogenicity assays are developed and deployed. The proposed NNEM strategy thus provides a generalizable framework for enrichment of datasets with previously collected assays indicative of species.

## Introduction

Pathogens can exhibit a diverse set of properties, such as antibiotic resistance, host cell adherence or host cell cytotoxicity. For example, a pathogenic strain might be toxic but not antibiotic resistant, or vice versa. Integrating data from multiple sources, each of which examines different properties, allows each dataset to provide information another source lacks^1^. Consequently, models aiming to predict pathogenic potential can be made more effective by integrating results from more than one phenotypic assay^2,3^. For these efforts, data integration is not trivial and the challenge is further compounded when researchers want to include data generated in prior experiments to enhance the analysis of current observations^4–6^. The prior datasets are often collected with a specific objective in mind (e.g., measure substrate utilization of a bacterium) and so often must be renormalized or harmonized to enable integration and downstream analysis with data from other experiments.

Integration of data from the Centers for Disease Control and Prevention (CDC) Special Bacteriology Reference Laboratory (SBRL) into new assays provides an excellent example of this dataset integration challenge. The SBRL dataset has been under construction since the 1950s and contains curated results of more than 30 biochemical assay data from 28,000 bacterial strains that cover approximately 500 species. Prior to the ubiquity of WGS, these assays were the standard method used to identify bacterial strains. The assays include tests to determine substrate utilization and catalytic activities. Given its size and assay diversity, we hypothesized that this dataset which encoded aspects of a strain’s identity could provide extra context for a model trained on hallmarks of pathogenicity to assess the threat posed by the bacteria. Here, context refers to the biochemical assays that were used to identify the strain. We recognize that there are newer, more precise techniques for species identification, such as sequencing or mass spectrometry analysis. Our goal here, however, was to take a large, curated prior dataset that was simple to parse but very noisy to see if the additional context about a strain would be useful before undertaking more complex tasks of sequencing or mass spectrometry. The CDC SBRL datasets associated the test results with a unique identifier for each strain, and the only information available for integration of the assays was the species of the bacteria. Therefore, to integrate this dataset with bacterial strains from our new experimental data that measured hallmarks of pathogenicity is non-trivial as the new experimental data represents a different set of tests as well as a different set of strains, with no common strain IDs. We therefore had to find a way to summarize the CDC SBRL data in a manner that would be useful to our machine learning algorithms trained with the pathogenicity assays.

**Figure 1:**
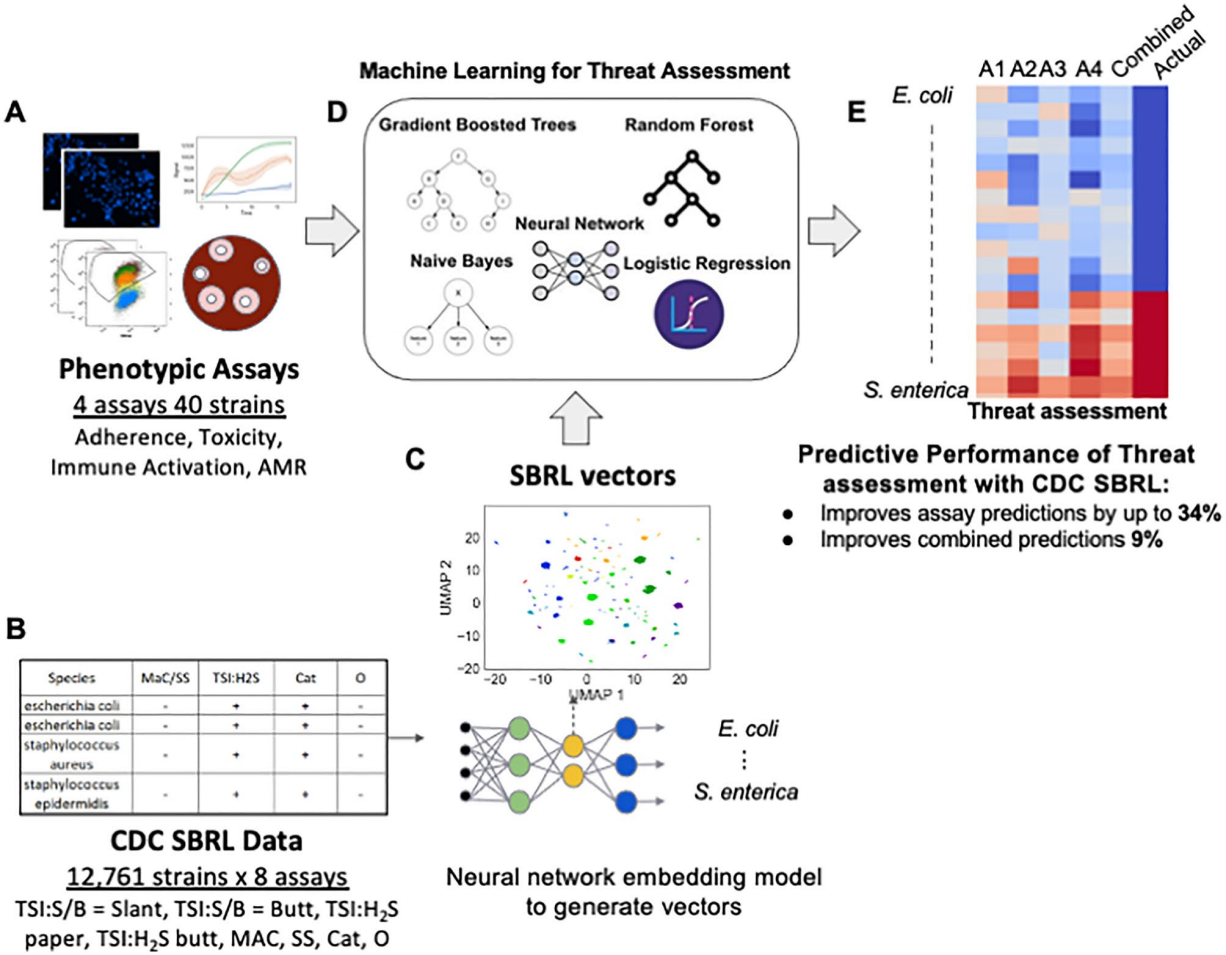
SBRL dataset of phenotypic assays for various strains cannot be easily integrated with other data. (**A**) Data for hallmarks of pathogenicity. (**B**) The CDC SBRL dataset contains additional phenotypic assays that focus on identifying bacterial strains. (**C**) A neural network embedding model to generate bacterial vectors at the species level so the vectors can be integrated with the pathogenicity assays. (**D**) Each assay integrated with the SBRL vectors are input to ML models to make predictions on bacterial pathogenicity. (**E**) Evaluation of pathogenic of each bacterial strain from each assay and from combining assays. Red represents a pathogen and blue represents a non-pathogen. A1–A4: predictions from each assay. Combined: statistical ensemble of A1–A4. Actual: the actual label.

Our work presents a solution to integrate data from prior host toxicity, antimicrobial resistance (AMR), immune reporter, and host adherence assays (henceforth referred to as pathogenicity assays) with CDC’s SBRL data to determine bacterial pathogenicity. This integration enhanced predictions made by machine learning models trained only with pathogenicity assay data (Fig. 1A). We used a deep learning approach to embed CDC’s SBRL data (Fig. 1B) into a vector space. The vectors were thought of as a new representation for the identification of a strain using data from biochemical assays suitable for machine learning. We then designed an integration technique with data collected from new assays to provide a significant improvement to prediction of pathogenic potential of bacteria with models trained with the pathogenicity assays. We trained a set of machine learning (ML) models for each pathogenicity assay and then combined predictions across assays for a final pathogenicity call (Fig. 1D). We hypothesized that additional context from bacterial species provided by the CDC SBRL dataset would enhance the ML models, but there was no easy way to integrate the data with the pathogenicity assays directly. To address this issue, we developed two methods to integrate the two datasets.

The first method computed a percent positive signal (pps) per species in the CDC SBRL data and the second method used a deep learning model referred to as a neural network embedding model (NNEM). The NNEM generated mathematical vectors representing bacteria across species from the CDC SBRL dataset (Fig. 1C) that could be integrated with data from the pathogenicity assays. The vectors were then integrated with the pathogenicity assays to be used as input into the ML models (Fig. 1D) to assess bacterial pathogenic potential (Fig. 1E). The vectors serve as a “prior” for the ML models to provide additional context to the data from the pathogenicity assays. By including the SBRL data, we observed an improvement up to 34% in the accuracy for the immune activation assay and a 9% improvement in the overall accuracy when all the assay predictions were combined. Performance improvement was assessed by comparing these results to a set of models that were only trained with pathogenicity assays.

The strategy to integrate heterogeneous data presented here has broader impacts in three aspects. First, the generated vectors can be integrated with other pathogenicity assays as long as the species names are available. Second, although our goal was to predict pathogenicity, the same methods can be applied to other tasks such as phenotypes to characterize bacterial metabolism or enzymatic activity. For example, researchers may be interested in bacterial vectors associated with metabolism rather than vectors associated with pathogenicity. Third, this strategy has the potential to be used in other fields of life sciences where terminology or resolution of terminology are updated over time like immune cell subsets.

## Results and discussion

### A vector representation of the SBRL assays that preserves species discrimination

The CDC SBRL dataset contains more than 30 different assays that include tests to determine substrate utilization and catalytic activities. Prior to the advent of DNA sequencing, these phenotypic assays were the only method available for bacterial species identification among bacteria that had similar gram staining and colony morphology. The dataset was narrowed down to focus on eight assays that had measurements listed in them for at least 80% of the strains (Table 1).

**Table 1:** SBRL assays designated as relevant for threat assessment used in this work.

Others	Possible outcome	Threat relevance
MaC/SS = SS (Salmonella Shigella agar)	−/+	High
MacC/SS = MacC (MacConkey agar)	−/+	High
TSI:H2S = paper (hydrogen sulfide production on lead acetates paper)	−/+	Medium
TSI:H2S = butt (hydrogen sulfide production in butt)	−/+	Medium
Cat (catalase)	−/+	Medium
O (oxidase)	−/+	Medium
TSI:S/B = Slant (color change—slant)	A,a,K,k,NC	Medium
TSI:S/B = Butt (color change—butt)	A,a,K,k,NC	High

*A* acid, *a* slight acid, *K* Basic, *k* slight basic, *NC* no change.

**Figure 2:**
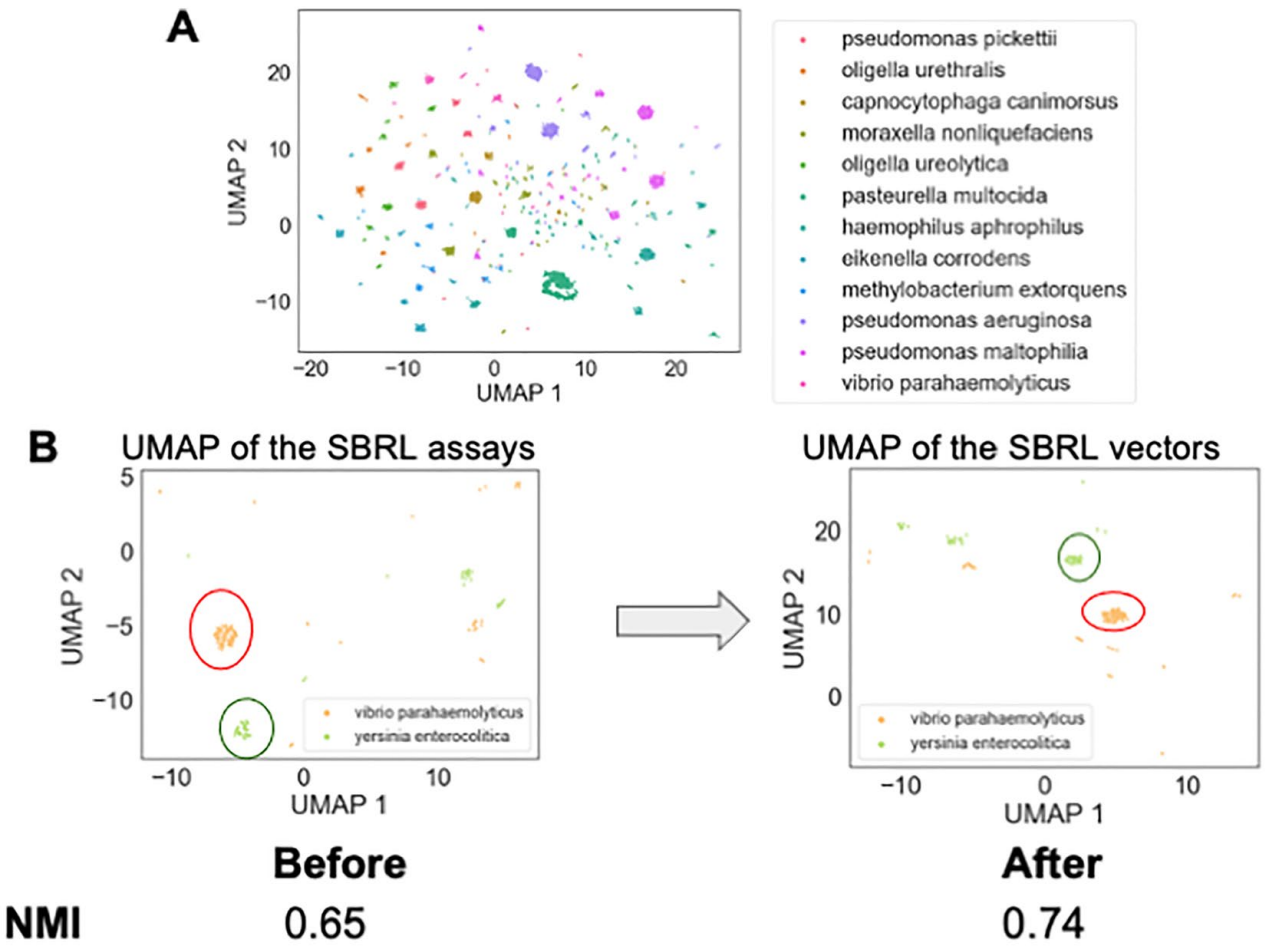
Exploratory data analysis discovered that the SBRL dataset discriminate between different bacterial species. (**A**) 2D UMAP was performed on the SBRL assays followed by k-means clustering to provide the bacterial samples cluster labels. Every point in the plot is a bacterial sample. The points form groups in the UMAP, suggesting that the SBRL assays can aggregate similar bacteria together. The colors in the figure are the k-mean labels. (**B**) The neural network model pushes the samples from the same bacteria species closer together. An example output of two species, Vibrio parahaemolyticus and Yersinia enterocolitica, are shown in the UMAP before and after training to show clusters are refined by the model. We quantified how well the samples from the same species are clustered together before and after the training and found the normalized mutual information went from 0.65 to 0.74.

To determine if these eight assays can differentiate between various types of bacteria, a Uniform Manifold Approximation and Projection (UMAP) dimension reduction was performed to visualize the dataset (Fig. 2A). Every point in the plot was a bacterial strain. The clusters that were formed based on the results from the selected eight assays belonged to bacteria with the same species names, suggesting the machine-learning approach to use the SBRL results to aggregate similar bacteria together can recapitulate the observations of human microbiologists that were made over the course of decades. The subset of assays that the computer scientists used maintained discriminative power across species.

The next challenge was to develop a vector representation for the assays that would be useful to downstream machine learning models. Two solutions were investigated to address this limitation, both of which integrated the data based on species identification. The first method computed the percent of species that have a positive signal from the assay, henceforth referred to as pps (percent positive signal). PPS was considered as a positive control, as it enhanced the pathogenicity assays with the SBRL dataset but did so without the use of machine learning. The second method used a neural network embedding model (NNEM) to create bacterial species vectors using the data from the biochemical assays, henceforth referred to as vectorization. Given we only used data from eight assays and wanted to remain comparable to the PPS, we did not choose to change the dimensionality from eight. The model simply transformed the representation of the eight assays into an eight dimensional vector per species. This process involved as input the various bacterial strains and their biochemical characteristics into NNEM, then asking the model to predict the species name for each strain based on the assay. As Fig. 2A showed, this should be possible by the model. The architecture of the neural network model is shown in Supplementary Fig. 3. As the model was trained to predict the species name for each strain, it created distinct vectors for each species and these new distinct vectors represented the species for downstream analyses. This learned vector representation of the SBRL biochemical assays was then integrated into our pathogenic models at the species level. In a sense, this approach combined very old data with very new algorithms to enhance the predictive power of machine learning models trained to predict pathogenic potential. We observed that after the NNEM training, the *Vibrio parahaemolyticus* strains and *Yersinia enterocolitica* strains from the initial panel of 40 formed tighter clusters (Fig. 2B). We quantified how much the NNEM helped the strains that belong to the same species cluster together and found an improvement in the normalized mutual information^7^, a metric used to measure how well groups cluster, from 0.65 to 0.74. It should be noted that we do not claim that the NNEM can distinguish between strains perfectly, as can be seen from the normalized mutual information scores. Namely, if it was perfect, NMI = 1. We instead used the vectorization to provide a species prior for our machine learning models trained only on pathogenicity assays to benefit from the additional context.

### Integrating SBRL data with custom pathogenicity assays using a neural network embedding model (NNEM)

Previously, the PathEngine platform^2^ was developed to evaluate results of four phenotypic assays that measure pathogenic potential of a blinded set of 40 bacterial strains. These four pathogenicity assays would reasonably be expected to associated with bacterial pathogenicity due to known biological mechanisms^8,9^. The host immune activation assay detected activation of NF-κB signal Jurkat T lymphocytes to capture presence of pathogen-associated molecular patterns (PAMPs)^10,11^. The AMR assay was used to discover antibiotic resistance, providing an indication whether any instance of infection could be efficiently treated^12^. The host adherence assay measured the ability of bacteria binding to host cells, a crucial step for pathogens to establish an infection^13,14^. Lastly, the host toxicity assay detected host cell death induced by the bacteria to measure the cytotoxicity of these strains^15,16^. The data produced by the assays were used to train ML models to predict a strain’s pathogenic potential from these properties. Traditionally, an expert would review the data and make a pathogenic call based on their interpretation of the data. Here, the model learns the features from each assay and then combines those features into an ensemble model that makes a pathogenic call. The model from each assay as well as the ensemble is compared to the “friend or foe” designation provided by NIST. Details can be found in our prior work^2^. The CDC SBRL dataset contains some of the same species as the bacteria used for PathEngine analysis. It was therefore hypothesized that by integrating the SBRL data with the results of the four pathogenicity phenotypic assay data, the models would have more context about each species and achieve better performance.

However, the SBRL data was not easily integrated with the results of the pathogenicity assays, since none of the actual strains tested for pathogenic potential were present in the SBRL dataset. The two representations described in the previous section were then integrated at the species level, rather than actual strains. In other words, every strain was supplemented with SBRL data that was represented through pps or vectorization. Having established two ways to integrate the SBRL biochemical data with results from the pathogenicity assays, we then performed three tests to evaluate if, and how much, the integration of the SBRL biochemical data impacted the ML results. A total of 22 bacterial strains that belong to 14 unique species were enriched with the SBRL data based on the species names. Note that we had 40 strains to use without integrating with the SBRL data but only 22 left after the integration as the remaining species were not in the SBRL dataset (Supplementary Table 1). With many fewer strains for training and testing, the accuracy of the ML models to predict pathogenic potential was expected to be lower than we had in the original PathEngine paper^2^, as smaller dataset sizes are generally understood to result in lower performance for this sort of model. For each assay, we tested a model with 10× cross validation that used either (1) the pathogenicity assays only, (2) the pathogenicity assay combined with the pps or, (3) the pathogenicity assay combined with the vector representation created by the NNEM. These models were used to test how well the PathEngine predictions matched the pathogenicity designations provided by NIST. We used “balanced accuracy” as the metric to ensure that the performance was not biased towards the majority class and henceforth refer to this metric as “accuracy”. The possibility that the observed prediction improvement was due entirely to the removal of less well-understood bacterial strains from the analysis was precluded by the fact that a control condition of prediction from assay without SBRL vectors, as well as with SBRL pps. Any and all improvement can thus be attributed to the vector representation we developed.

**Figure 3:**
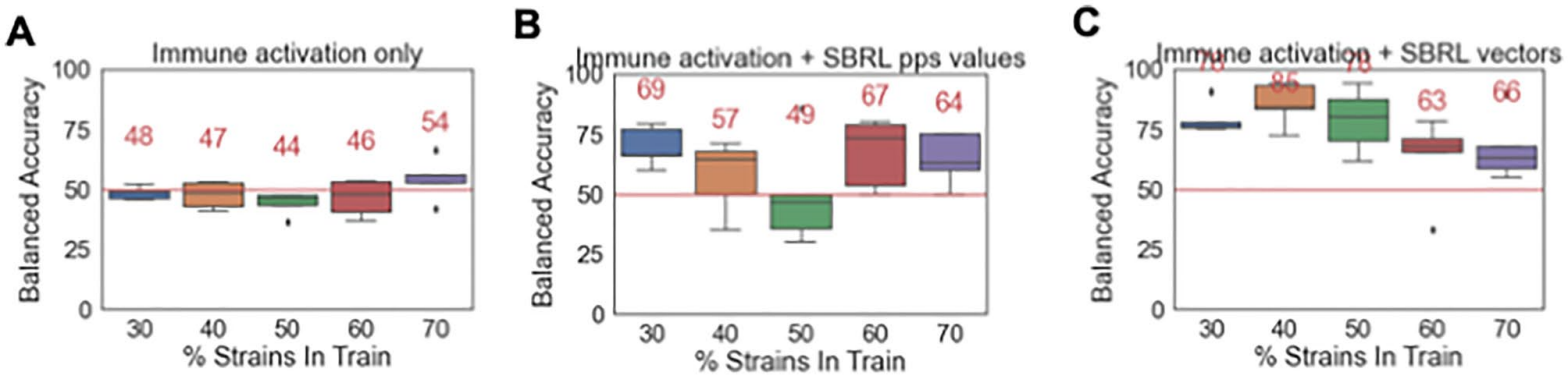
Incorporation of information from SBRL enhanced the predictions of pathogenic potential of the immune activation assay up to 34%. (**A**) Ten-fold cross validation of an ML model with an A. immune activation assay data alone, (**B**) the percent positive signal (pps) and (**C**) NNEM of SBRL data. The results went from 51%, 75%, to 85%, balanced accuracy respectively.

For the immune activation assay, adding the pps increased the ML accuracy up to 24% (Fig. 3A,B). When the vector representation were used instead of the average values, the accuracy improved from 51 to 85% (Fig. 3A,C).

**Figure 4:**
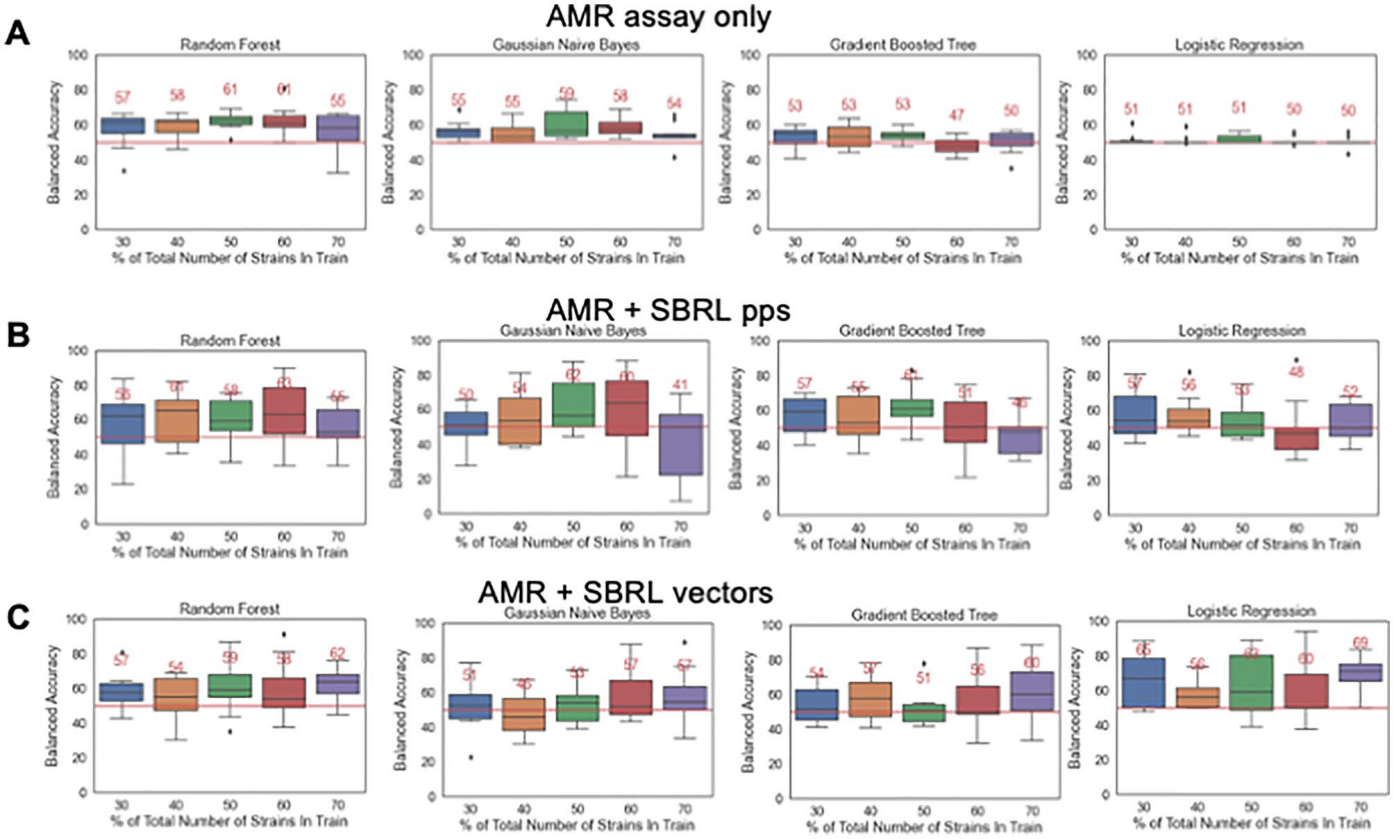
Incorporation of information from SBRL enhanced the predictions of pathogenic potential of the AMR assay up to 8%. (**A**) Ten-fold cross validation of an ML model with an A. AMR assay data alone, (**B**) the percent positive signal (pps) and (**C**) NNEM of SBRL data. The results went from 61%, 63%, to 69%, balanced accuracy respectively.

**Figure 5:**
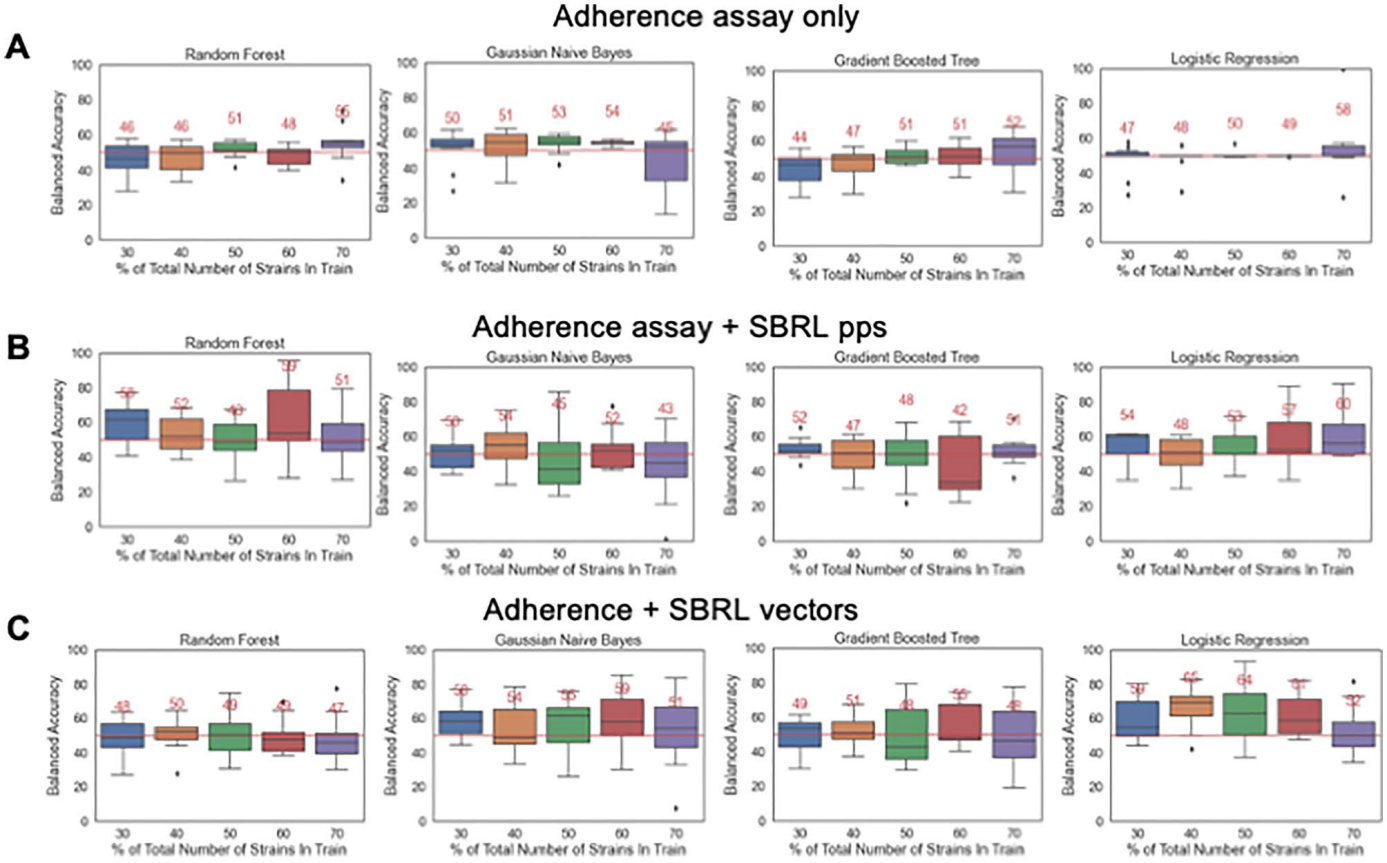
Incorporation of information from SBRL enhanced the predictions of pathogenic potential of the adherence assay up to 7%. (**A**) Ten-fold cross validation of an ML model with an A. adherence assay data alone, (**B**) the percent positive signal (pps) and (**C**) NNEM of SBRL data. The results went from 58%, 60%, to 65%, balanced accuracy respectively.

For the AMR assay, pps increased the accuracy by 2% (Fig. 4A,B) and the vectors improved the accuracy by 8% (Fig. 4C). For the adherence assay, pps increased the accuracy by 2% (Fig. 5A,B) and the vectors improved the accuracy by 7% (Fig. 5C). The toxicity assay is the only exception where the performance decreased when the SBRL representations were included (Supplementary Fig. 1A–C).

In order to investigate the cause for decrease in performance of the toxicity assay predictions, all the predictions were grouped into four prediction classes (“−” predicted as “−”, “−” predicted as “+”, “+” predicted as “−” and “+” predicted as “+”). Namely, each bacterial observation was classified as either non-pathogenic (“−”) or pathogenic (“+”). DAPI signals from the toxicity assay showed that the host cell death induced by the bacteria can be distinguishable between different prediction classes (Supplementary Fig. 2A). After integrating the DAPI signal and the SBRL assays, we observed that the signals were masked by the presence of the SBRL assays and stayed flat throughout the time course. The assays were not as distinct between different classes as before (Supplementary Fig. 2B). Similar observations were seen when the SBRL vectors were incorporated (Supplementary Fig. 2C).

### Ensemble prediction of pathogenic potentials is enhanced in the presence of the SBRL vectors

As each assay reveals different aspects of bacterial pathogenicity^8,9^, we combined predictions from the best performing model from each of the four assays to make a final threat assessment call. Using the models trained without using the SBRL vectors for the ensemble, we achieved accuracy of 70%, precision of 86%, recall of 73% and F1 of 79%. When the SBRL vectors were included, the ensemble performance achieved accuracy of 79%, precision of 90%, recall of 82% and F1 of 86% (Table 2). These results confirmed that adding the SBRL data provided useful context about the bacterial species for the ML models and thus improved the pathogenicity predictions.

**Table 2:** Performance of aggregating predictions from the four assays using the model trained with and without SBRL vectors.

	Accuracy	Precision	Recall	F1
4 assays	70	86	73	79
4 assays with SBRL vectors	79	90	82	86

### Threat designations of the SBRL vectors based on literature and the models show consistent trends

To understand which SBRL assays were useful for the model predictions, we annotated each assay based on literature review and also quantified the assay importance by data-driven approaches. The assays are listed and annotated with their relevance for threat assessment in Table 1. For the data-driven approaches, we first examined the signals for the four prediction classes. If the “− as −” (non-pathogens predicted to be non-pathogens) and “+ as +” (pathogens predicted to be pathogens) had dramatically different signals, it suggested that the assay is likely useful for threat assessment.

**Figure 6:**
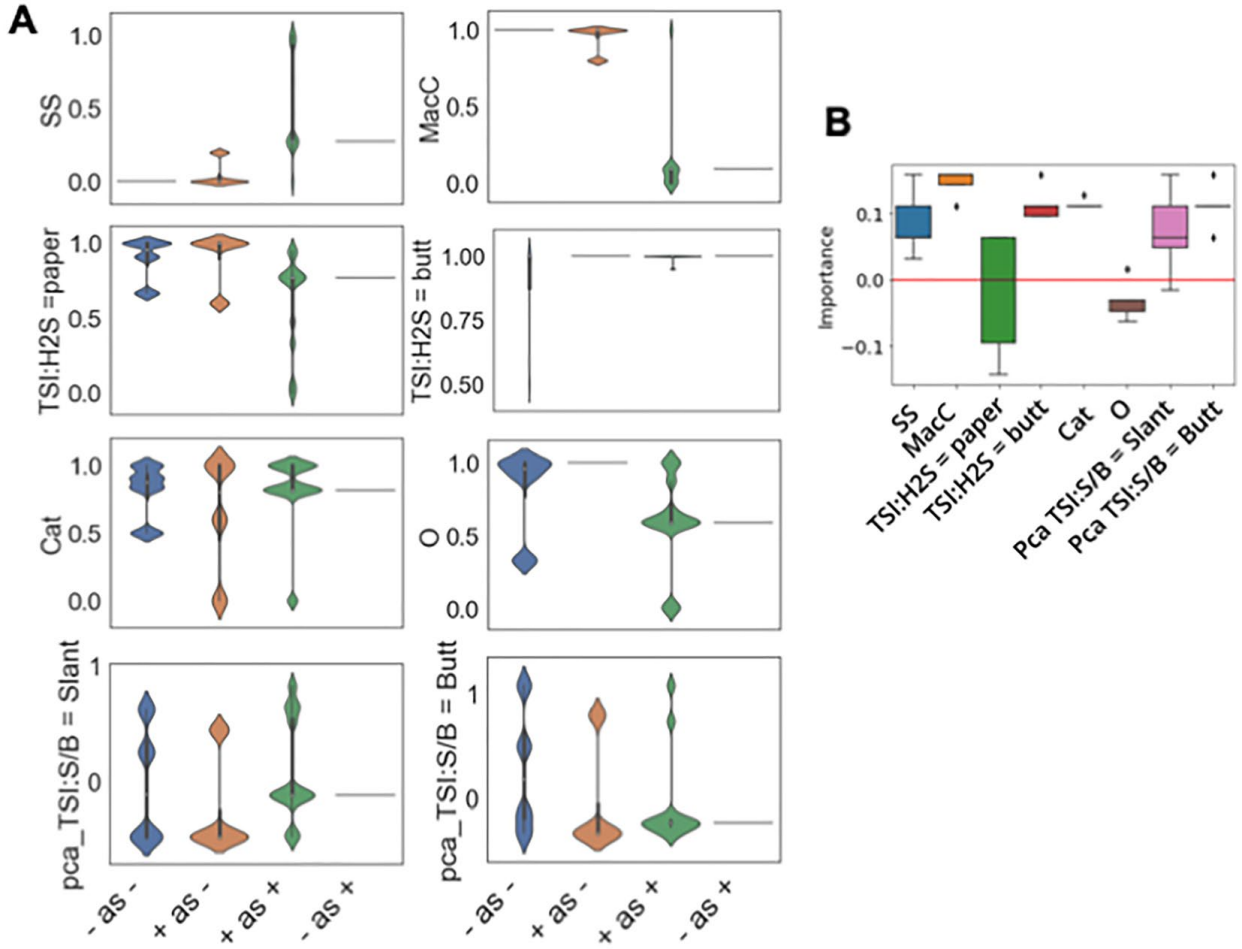
Comparison of threat designations of the SBRL assays based on literature and the contribution determined by the models. **(A**) Data-driven qualitative assessment of threat relevance of the SBRL assays based on ML predictions. Non-pathogenic strains annotated as “−” and pathogenic strains as “+”. The predictions belong to 4 groups: “− predicted to be −” , “− predicted to be +”, “+ predicted to be −” and “+ predicted to be +”. SS, MacC are the most useful assays as their “− predicted to be −” and “+ predicted to be +” groups are differentiable. (**B**) The quantitative measurement of the assay contribution by determining the changes in performance when each assay is dropped one by one. If an assay is dropped and the accuracy decreases, the assay gets a positive importance score and vice versa.

Consistent with the literature designation, MacConkey Agar (MacC) and Salmonella Shigella (SS) Agar are most relevant for threat assessment as they have the most pronounced difference between the “− as −” and “+ as +” classes (Fig. 6A). This is consistent with established microbiological understanding. Specifically, growth on MacConkey agar and SS agar are highly associated with pathogenicity, because most Enterics will grow on these agars. These assays are what have always been used to separate coliform bacteria from other similar bacteria. The re-discovery of these markers by computer scientists with no training in microbiology is a testament to the usefulness of a data-driven approach. It gives us confidence that heretofore unrecognized markers of pathogenicity will be similarly detectable. Supplementary Table 1 lists all the species used in this assay. Details of these strains and associated tags have been described previously^2^. The rest of the assays used were not as distinguishable as MacC and SS between the “− as −” and “+ as +” classes but did show noticeable differences to be considered as assays useful for threat assessment as supported by the literature (Table 1). To quantify the importance, we performed drop-assay tests where we dropped one assay at a time and compared the change in the model performance to the baseline where no assay was dropped. The change in the performance quantified the importance of the assay. We found the majority of the assays have positive importance for predicting pathogenicity with the exception of lead acetate paper (TSI:H_2_S = paper) and oxidase tests (O) (Fig. 6B).

## Conclusion

In this work, we successfully integrated the CDC SBRL data with phenotypic assay by using NNEM to generate mathematical vector representations of bacterial species. The vectors improved the prediction accuracy of threat assessment up to 34% for the pathogenicity assays and achieved 9% improvement in the accuracy in the ensemble prediction. Note that by integrating with the SBRL data, we had a total of 22 strains to perform train-test split, meaning that it was a rather small data size for ML models, yet we achieved the best accuracy of 79%. In addition, we determined the assay importance of the SBRL data and found that it was in general agreement with the literature. The main contribution of this work was to show that inclusion of additional context from a prior dataset supplemented our models that were trained only on pathogenicity assays to assess pathogenic potential. Going forward, we expect more advanced datasets, such as those provided by WGS or mass spectrometry to only enhance these results. This method works best where the assays are processed into distinct dimensions of the strain or species under study. In the case of WGS or mass spectrometry, this could be a strain described by particular genes, their differential expression, the abundance of a protein, or other known chemical product.

Although we present a solution to integrate data, there is a limitation in this approach. The vectors work because it provides context about the bacteria but if the context has mixed information such as the strains within the same species are highly divergent and can be either strong pathogens or non-pathogens, the vectors are not likely to give useful information about the pathogenicity of the species.

Finally, we expect this approach to have a broader impact in life sciences as changes in terminology is common in the field. Researchers would likely need to first determine whether the vectors provide information at the sufficient resolution. For example, if we had generated vectors at the genus level, this approach would likely not work as the bacteria could be highly divergent at the genus level. Nonetheless, this strategy provides a solution to join separate sources and improve phenotypic classification.

## Materials and methods

### The CDC SBRL data for generating bacterial vectors

The assays we used from the SBRL dataset included MacConkey agar (MacC), Salmonella Shigella agar (SS), triple sugar iron agar (TSI) with color change in the aerobic areas of the slant (TSI:S/B = Slant), TSI with color change in the anaerobic butt area of the slant (TSI:S/B = Butt), TSI with hydrogen sulfide production on lead acetate paper (TSI:H_2_S paper), TSI with hydrogen sulfide production in butt (TSI:H_2_S butt), catalase (Cat), oxidase (O). The assays were performed as described previously^17^.

### Custom phenotypic assays to measure pathogenicity

The four phenotypic assays used to characterize bacterial pathogenicity were described in our previous paper^2^. In brief, the assays were host cell adherence with the A549 cell line, host cell toxicity with the THP1 cells, immune activation with the Jurkat NF-kB reporter cells and antibiotic resistance with ampicillin, kanamycin, tetracycline, chloramphenicol, polymyxin B and ceftazidime.

### Neural network embedding model

Each of the SBRL test results was first encoded as numbers. For example, for the MacC test, it was either negative or positive so they were encoded as 0 or 1. For the TSI:S/B = Slant test, the possible results were A (strong acid), K (strong base), NC (no change), a (weak acid), NG (no growth) and k (weak base). Each result was encoded into a number from 0 to 5. Each assay was then fed into an input layer of the neural network. The input layers were connected to an embedding layer. All of the embedding layers were concatenated and flattened. The model then generated a dense layer to predict the species name of the input bacterial samples. A total of 1630 bacterial strains were used to train this model and 408 strains were used to test the model with no strain overlap in the train and test and the test accuracy was 0.895. The detailed architecture of the model is shown in the Supplementary Fig. 3. After the model was trained, the 8-D flatten layer prior to the output layer was retrieved and used as the bacterial vectors (*E*_*post*_) to integrate with custom pathogenicity assays. The same SBRL data were embedded in the same manner without training the model and this embedding was used for comparison, called pre-training embedding (*E*_*pre*_). The improvement of clustering the same species together by the NNEM was quantified by the method described below.

- Species labels (S): species names for each bacterial strain.
- NMI: function for calculating normalized mutual information
- KMeans: k-means clustering to obtain cluster labels for each bacterial strain
- Pre-training cluster (*C*_*pre*_) = KMeans(*E*_*pre*_), k: number of species
- Post-training cluster (*C*_*post*_) = KMeans(*E*_*post*_), k: number of species
- NMI for before training: NMI(S, *C*_*pre*_)
- NMI for after training: NMI(S, *C*_*post*_)

### UMAP of the SBRL data

All the UMAP figures were generated with the Python umap-learn package version 0.5.1. The parameters to generate the UMAP plots were n_neighbors = 2 and min_dist = 0.8. UMAP of the SBRL assays were 2D UMAP plots of the embedded assays without training the NNEM. UMAP of the SBRL vectors were UMAP of the trained embedding (vectors described in the “Neural network embedding model” section).

### ML models to predict bacterial pathogenicity

For the immune activation assay, we used a custom deep learning model as there were thousands of cells for each sample and the large sample size allowed deep learning models to perform well. The model architecture is shown in the Supplementary Fig. 4A. We used a custom loss function that is a combination of the binary cross entropy and entropy loss (Supplementary Fig. 4B). The entropy loss measures how uniform the predictions are within the same species as all the observations from a single species should in theory get the same predicted label. If the predictions are divergent with almost equal proportions of 0 s and 1 s, the entropy loss would be large and vice versa. The deep learning model was implemented with TensorFlow 2.6.0. For the host adherence, host toxicity and AMR assays, we used non-deep learning ML models to avoid overfitting because these assays have data in the range of the hundreds to low thousands. The models were random forest classifier (n_estimators = 361, max_features = auto, criterion = entropy, min_samples_leaf = 13), Gaussian Naive Bayes classifier (default for all parameters), Gradient Boosted Tree classifier (learning_rate = 1, loss = deviance, max_depth = 10, max_features = auto, n_estimators = 100) and Logistic Regression (penalty = l1, C = 0.1, solver = liblinear). All the non-deep learning models were implemented with scikit-learn 0.24.1.

Each model was tested in three ways: The first set only used the pathogenicity assay alone. The second set combined the pathogenicity assay and the SBRL assay pps. These values came from converting all of the possible SBRL test results into columns. For example, MacC had two possible test results: − and + so there were two columns for MacC: MacC− and MacC+. We aggregated across the strains from each species to obtain the proportions for MacC− and MacC+. *Escherichia coli* has MacC− value as 0.091 (9.1% of the *E. coli* strains have MacC test as +) and MacC+ value as 0.909 (90.9% of the *E. coli* were MacC−) and the two values would always sum up to 1. The third set was a combination of the pathogenicity assay and the SBRL vectors.

For each model, we swept the sample size (22 strains) from 30 to 70% of the bacterial strains to determine the number of samples needed to achieve the best performance and repeated each train-test-split 10 times. When a strain was in the train set, all of the data points that belonged to the strain would be in the train set so this ensured that the model was robust to predict pathogenicity for unknown strains.

### SBRL assay importance quantification

Each assay was transformed into ratios for each possible test result as described in the “ML models to predict bacterial pathogenicity” section. For the qualitative assessment shown in Fig. 6A, we annotated pathogens as “+” and non-pathogens as “−”. The normalized values were plotted for four prediction groups: “− predicted to be −” , “− predicted to be +”, “+ predicted to be −” and “+ predicted to be +”. For tests with binary outcomes such as MacC, SS, TSI:H_2_S = paper, TSI:H_2_S = butt, Cat and O, we only plotted one of two columns as the values for one column would simply be the 1-values for the other column. For TSI:S/B = Slant and TSI:S/B = butt, there were multiple test outcomes so multiple values. To simplify the visualization, we performed principal component analysis to obtain the principal component 1 (PC1) for these columns and showed the PC1 signals for the four prediction groups.

For quantitative assessment of the SBRL assay importance in Fig. 6B, we dropped each assay column one at a time to determine the changes in model performance. The control was when all the assays were present.

$$ {\text{Importance}} = 1 - \left( {\text{model accuracy/control accuracy}} \right) $$

### Toxicity assay signal before and after integrating with the SBRL data

The three sets of the toxicity assay were visualized by plotting them against the time points. For the toxicity assay alone, the min–max-scaled DAPI signal was shown as it represented the death induced by the bacteria. The toxicity assay was then joined with the SBRL pps and the SBRL vectors. The PC1 of the integrated datasets were plotted against the time points.

## Supplementary Information

Supplementary Information.

## Data Availability

All analyses are available on github: https://github.com/netrias/PathEngine/tree/master/notebooks/CDC_SBRL. Phenotype assay data used in the publication are also available in the repository. However, due to the sensitive nature of the CDC's SBRL dataset, it is available upon request to the Center for Disease Control, Special Bacteriology Reference Laboratory. They can be reached by email at SBRL@cdc.gov. The SBRL needs to track all parties interested in using the data and would like to minimize any potential for misinterpretation or attribution of the data. All code and results of using that data, however, is available in the Github repository linked. To re-run these analyses, you will be required to reach out to the CDC to retrieve the SBRL data and change the path in the notebooks to that path.
