# Supplementary material for: Neural network based integration of assays to assess pathogenic potential
Source: Sci Rep. 2023 Apr 13;13:6021. doi: 10.1038/s41598-023-32950-5 (PMC10102301; doi:10.1038/s41598-023-32950-5)
Supplement: Supplementary file 1 — Supplementary Information. [file 41598_2023_32950_MOESM1_ESM.pdf]

Neural Network Based Integration of Assays to Assess Pathogenic Potential

Mohammed Eslami[a*], Yi-Pei Chen[a], Ainsley C. Nicholson[b], Mark Westona, Melissa Bell[b], John R. McQuiston[b], James Samuel[c], Erin J. van Schaik[c], Paul de Figueiredoc,[d*]

[a]Netrias, LLC, 1162 Gateway Drive, Annapolis, MD 21409 USA

[b]Special Bacteriology Reference Laboratory, Bacterial Special Pathogens Branch, Division of High-Consequence Pathogens and Pathology, Centers for Disease Control and Prevention, Atlanta, Georgia 30333, USA

[c] Department of Microbial Pathogenesis and Immunology, Texas A&M Health Science Center, Bryan, TX 77807

[d] Department of Veterinary Pathobiology, Texas A&M University, College Station, TX 77843

The findings and conclusions in this report are those of the authors and do not necessarily represent the official position of the Centers for Disease Control and Prevention (CDC). The mention of company names or products does not constitute endorsement by CDC.

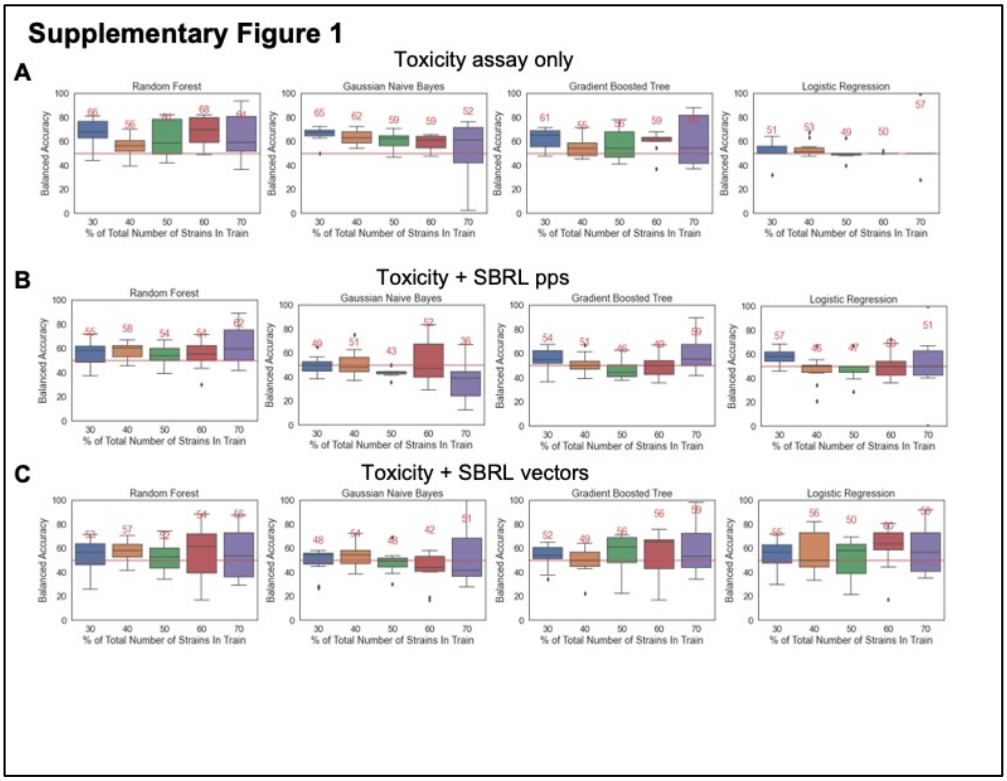

Supplementary Figure 1 Incorporation of the SBRL pps values or vectors did not improve the performance of the toxicity model. A. We trained and tested ML models with the toxicity assay to predict whether a bacterial strain is pathogenic or not. The best balanced accuracy was 68%. **B**. We included the pps values of the SBRL assay to the model and achieved 62% balanced accuracy. **C.** We included the SBRL vectors to the model and achieved 59% balanced accuracy.

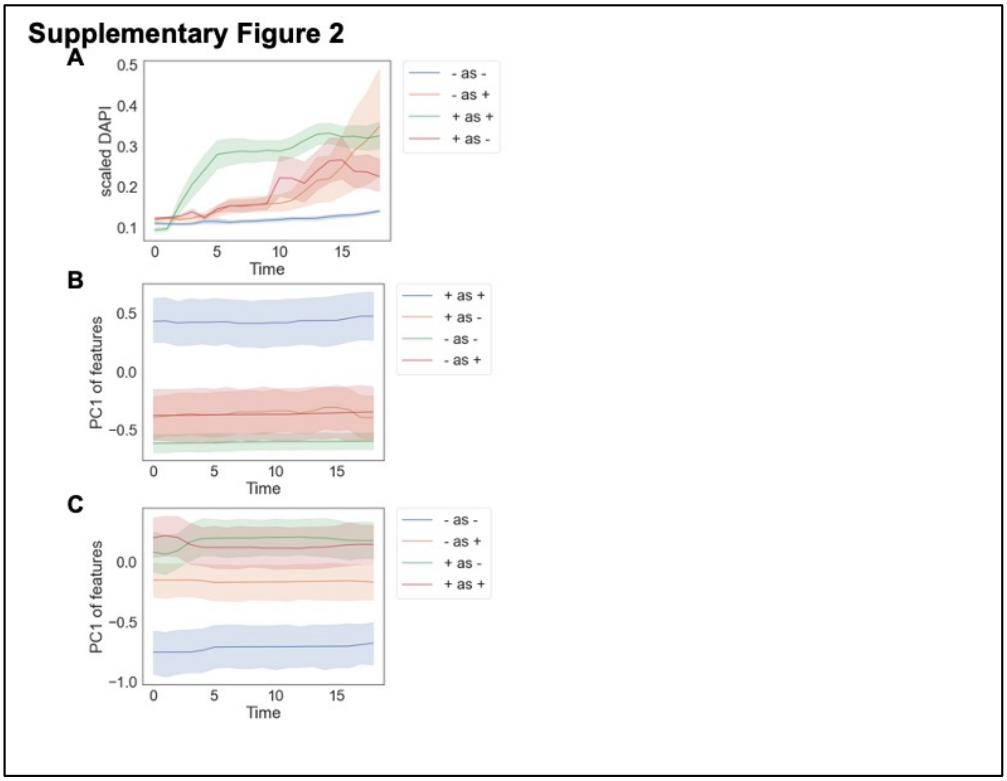

Supplementary Figure 2 The decrease in the toxicity assay performance is likely due to the discriminative power of the toxicity assay being masked by the SBRL data. A. Scaled DAPI signals represent host cell death. The signals are differentiable between the “- as -” (blue) and “+ as +” (green) groups. **B.** The Y-axis represents the principal component 1 (PC1) of the scaled DAPI signal plus the SBRL pps values. The scaled DAPI signal that would change throughout the time course is masked by the presence of the SBRL data as the signals stay flat throughout. **C.** Y-axis represents the PC1 of the scaled DAPI signal plus the SBRL vectors. Similar to B, the signal no longer changes throughout the time course.

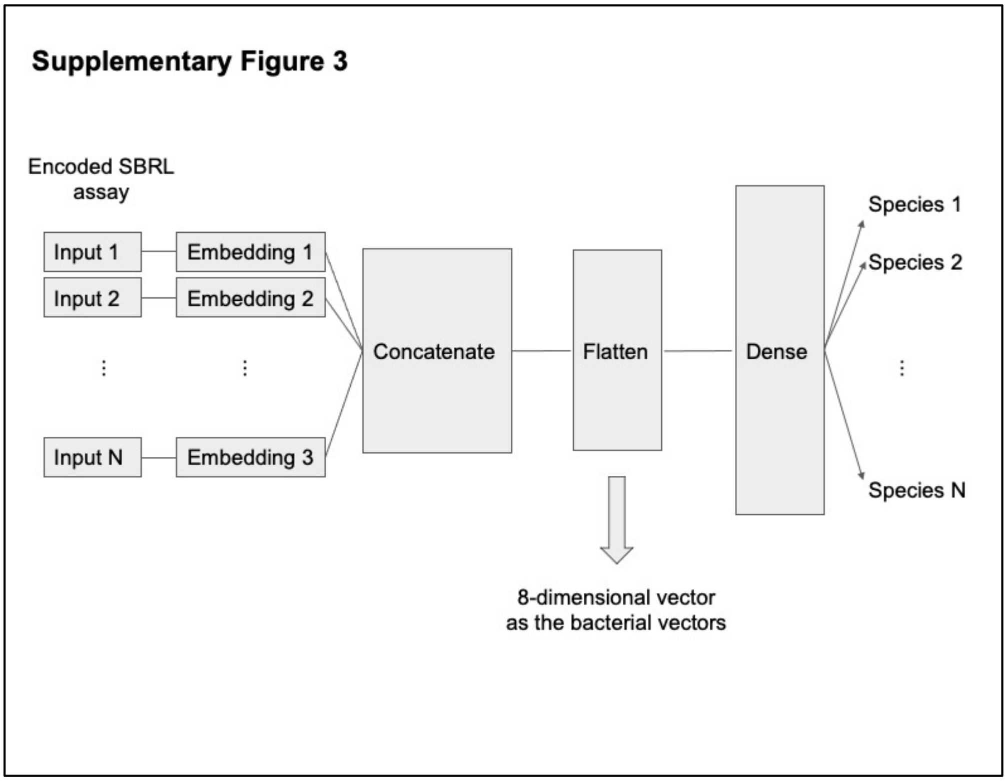

Supplementary Figure 3 Neural network architecture to obtain the SBRL vectors. All the SBRL assays used were categorical so they were encoded into numbers. Each assay type is an input for the model. The inputs are then passed through embedding layers and concatenated. The models are then trained to predict the bacterial species names. The second to the last layer was a 8-D flatten layer that is used as the bacteria vectors after the model training.

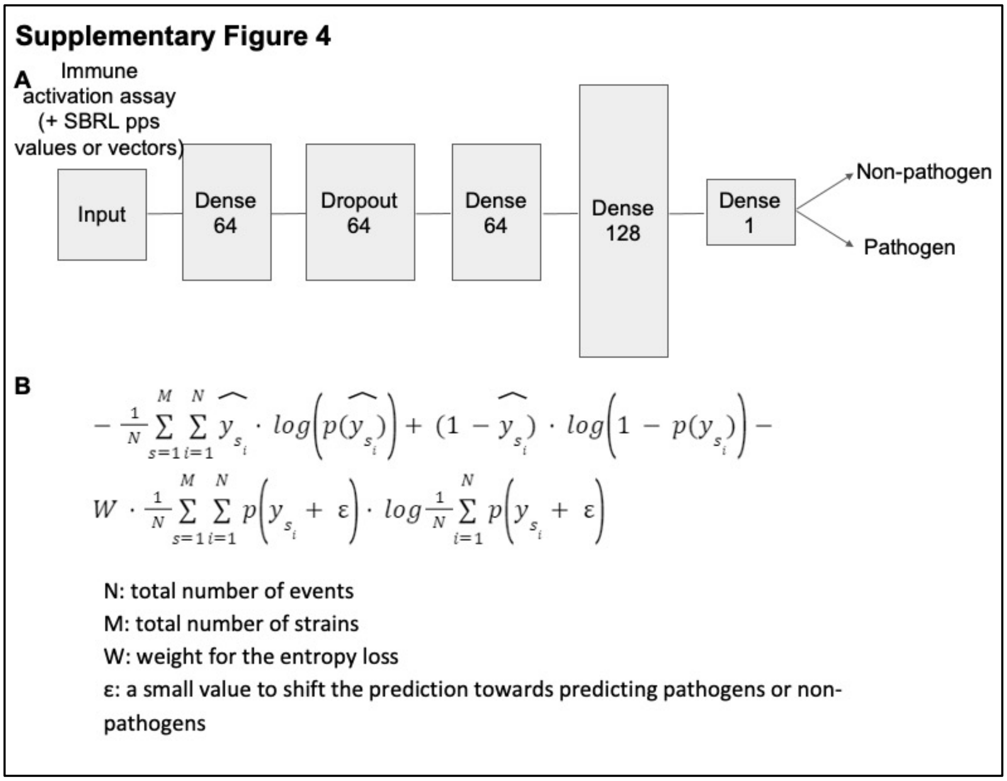

Supplementary Figure 4 Neural network architecture to predict data points in the immune activation assay. A. The immune activation assay (and the SBRL pps values or vectors) are input of the neural network. The output assigns each data point as a pathogen or a non-pathogen. B. The custom loss function used in A that combines the binary cross entropy (the first term) and the entropy loss (the second term).

Supplementary Table 1

Genus Species	Gram stain	Threat Level	Documented cytotoxins	Documented adhesins/ adherence	Documented immune evasion	Common/ rare/ emerging	Healthy/ opportunistic/ nosocomial	Reservoir
bacillus cereus	+	No Data	No Data	No Data	No Data	No Data	No Data	environmental/invertebrates
bacillus cereus	+	High	+	-	+	common	healthy	environmental
bacillus subtilis	+	No Data	No Data	No Data	No Data	No Data	No Data	No Data
clostridium perfringens	+	No Data	No Data	No Data	No Data	No Data	No Data	environmental/human commensal
clostridium tertium	+	No Data	No Data	No Data	No Data	No Data	No Data	No Data
corynebacterium amycolatum	+	Low	-	-	-	rare	opportunistic	skin commensal
corynebacterium diphtheriae	+	Low	+/-	+	-	rare/ emerging	opportunistic	environmental
enterococcus faecalis	+	Low	+/-	+	+	common	opportunistic/ nosocomial	gut commensal
enterococcus faecalis	+	No Data	No Data	No Data	No Data	No Data	nosocomial	GI tract
escherichia coli	-	No Data	No Data	No Data	No Data	No Data	No Data	human and animal commensal/environmental
escherichia coli	-	Medium	-	-	-	no reported infections	opportunistic	cultured from bird
escherichia coli	-	No Data	No Data	No Data	No Data	No Data	No Data	No Data
escherichia coli	-	High	+	+	+	common	healthy	GI tracts
escherichia coli	-	High	+	+	+	common	healthy	agricultural/ cattle
escherichia coli	-	High	+	+	+	common	healthy	agricultural/cattle
lactobacillus salivarius	+	No Data	No Data	No Data	No Data	No Data	No Data	No Data
listeria monocytogenes	-	High	+	+	+	common	healthy/opportunistic	environmental
proteus mirabilis	-	No Data	No Data	No Data	No Data	No Data	No Data	No Data
providencia stuartii	-	No Data	No Data	No Data	No Data	No Data	No Data	No Data
pseudomonas aeruginosa	-	High	+	+	+	common	opportunistic	environmental (water)
pseudomonas putida	-	Low	-	-	+/-	rare	opportunistic	environmental
serratia marcescens	-	No Data	No Data	No Data	No Data	No Data	No Data	No Data
serratia rubidaea	-	High	+	+	-	rare	opportunistic	environmental
staphylococcus aureus	+	High	+	+	+	common	nosocomial	skin commensal
staphylococcus aureus	+	High	+	+	+	common	healthy/opportunistic	skin commensal
staphylococcus epidermidis	+	Low	+/-	+	-	common	opportunistic	skin commensal
streptococcus pyogenes	+	No Data	No Data	No Data	No Data	No Data	No Data	No Data
yersinia enterocolitica	-	No Data	No Data	No Data	No Data	No Data	No Data	No Data
yersinia pseudotuberculosis	-	High	+	+	+	common	healthy/opportunistic	environmental

Supplementary Figure 4: Table of strains used in this work with associated properties and annotations. Annotations were provided by the National Institute of Standards and Technology. See [2] for details.
